# Cutaneous and noncutaneous features in patients with neurofibromatosis type 1: A retrospective cohort study including 644 patients

**DOI:** 10.1016/j.jdin.2024.03.010

**Published:** 2024-04-21

**Authors:** Kaya L. Curtis, Victoria De Barros, Samantha Jo Albucker, Yuqing Qiu, Shari R. Lipner

**Affiliations:** a Weill Cornell Medical College, New York, New York; b Tulane University School of Medicine, New Orleans, Louisiana; c Department of Population Health Sciences, Weill Cornell Medicine, New York, New York; d Department of Dermatology, Weill Cornell Medicine, New York, New York

**Keywords:** café au lait, cardiovascular, endocrine, freckling, neurofibroma, neurofibromatosis type 1, psychiatric, skeletal

*To the Editor:* Neurofibromatosis type 1 (NF1) is an autosomal dominant neurocutaneous disorder due to loss-of-function mutations in the tumor suppressor gene NF1.^1^ Up to 50% of NF1 cases are sporadic^2^ and variable expressivity contributes to phenotypic variability. Despite the broad range of systemic manifestations, few studies assessed comorbidity prevalence among NF1 cohorts. Therefore, we conducted a retrospective cohort study assessing the most common dermatologic and nondermatologic manifestations among patients with NF1.

Medical records of patients with NF1 at Weill Cornell Medicine 1979 to 2022 were reviewed for cutaneous findings and medical/psychiatric comorbidities. Descriptive statistics and χ^2^ tests were performed with *P* ≤ .05 (Supplementary Methods, available via Mendeley at https://data.mendeley.com/datasets/d5x7xzw9nd/1). This data set was used in an independent study.^3^

There were 644 patients with NF1 included, with mean age 29 years (Supplementary Table I, available via Mendeley at https://data.mendeley.com/datasets/d5x7xzw9nd/1). Overall, 41.7% of patients with NF1 had NF1 family history. The most common cutaneous findings were café au lait macules (CALMs) (99.1%), intertriginous freckling (92.7%), and cutaneous neurofibromas (62.1%) (Table I). The most common presenting signs were CALMs (91.5%), intertriginous freckling (36.8%), and plexiform neurofibromas (6.0%) (Table II).

**Table I:** Clinical features of patients with neurofibromatosis type 1 by organ system

Clinical features	Prevalence (%, *n*)
Cutaneous∗	
Café au lait macules	99.1% (550/555)
Intertriginous freckling	92.7% (455/491)
Cutaneous neurofibroma	62.1% (231/372)
Subcutaneous neurofibroma	57.8% (185/320)
Plexiform neurofibroma (superficial and deep)∗	66.3% (262/395)
Neurologic	
Hypotonia	13.4% (86)
Learning disability	13.0% (84)
Headache unspecified	7.8% (50)
Developmental delay	6.2% (40)
Migraine	5.0% (32)
Seizure	4.5% (29)
Abnormal gait	1.4% (9)
Back pain	1.2% (8)
Hydrocephalus	1.2% (8)
Stroke	1.2% (8)
Skeletal	
Scoliosis	17.4% (112)
Short stature	7.9% (51)
Macrocephaly	7.5% (48)
Leg length discrepancy	1.9% (12)
Leg bowing	1.4% (9)
Pes planus	1.2% (8)
Osteoporosis	0.9% (6)
Pseudoarthrosis	0.9% (6)
Sphenoid wing dysplasia	0.9% (6)
Psychiatric	
Attention deficit hyperactivity disorder	13.2% (85)
Anxiety	5.0% (32)
Depression	3.7% (24)
Autism spectrum disorder	1.6% (10)
Bipolar disorder	0.5% (3)
Obsessive compulsive disorder	0.3% (2)
Cardiovascular	
Hypertension	9.2% (59)
Hyperlipidemia	4.0% (26)
Hypercholesterolemia	1.7% (11)
Mitral valve prolapse	1.4% (9)
Moyamoya disease	0.6% (4)
Atrial septal defect	0.3% (2)
Endocrine	
Precocious puberty	3.4% (22)
Hypothyroidism	2.5% (16)

∗ Each patient’s chart was queried for the presence of café au lait macules, intertriginous freckling, cutaneous neurofibroma, subcutaneous neurofibroma, and plexiform neurofibroma. Patients who were not assessed for these features were not included in the denominator.

**Table II:** Presenting signs of patients with neurofibromatosis type 1

Presenting signs	Prevalence (%, *n*)
Café au lait macules	91.5% (335/366)
Intertriginous freckling	36.9% (135/366)
Plexiform neurofibroma	6.0% (22/366)
Cutaneous neurofibroma	5.2% (19/366)
Lisch nodule	4.1% (15/366)
Optic pathway glioma	3.8% (14/366)
Hypotonia	1.9% (7/366)
Macrocephaly	1.9% (7/366)
Presenting signs not reported	43.2% (278/644)

Overall, 19.7% (127/644), had psychiatric comorbidities, which were more common in White (22.4%) vs non-White (14.5%) patients (*P* = .04). Males (16.6%) vs females (10.1%) more often had attention deficit hyperactivity disorder (*P* = .01) and females (5.9%) vs males (1.3%) more often had depression (*P* = .002).

Skeletal abnormalities were present in 29.8% (192/644), with no difference in sex prevalence (*P* = .07). The most common skeletal findings were scoliosis (17.4%), short stature (7.9%), and macrocephaly (7.5%). Cardiovascular and endocrine abnormalities were present in 25.0% and 9.2%, respectively.

We found that ∼60% of NF1 cases were sporadic and almost all patients had CALMs. Similar to our findings, in a retrospective cohort study^4^ of 1102 patients with NF1, only 45.3% of patients had NF1 family history, with CALMs, intertriginous freckling, and neurofibromas present in 96.5%, 90.0%, and 78.1%, respectively.

We found that depression was more common in females vs males. Similarly, in a cross-sectional survey study^5^ of 498 patients with NF1, level of depressive symptoms by Center for Epidemiologic Studies Depression scale was higher among women (21.7) vs men (17.2) (*P* = .002). Higher prevalence of psychiatric diagnoses in white patients in our cohort may be due to mental health care underutilization or limited access among non-White patients.

We demonstrated that ∼1/3 of patients had skeletal abnormalities, similar to a retrospective cohort study^6^ of 32 pediatric patients with NF1 reporting 37.5% prevalence of skeletal changes, most commonly scoliosis (15.6%) and bowing (12.5%).

Limitations include retrospective and single-center design. Clinical detail for each case was inconsistently reported. CALM numbers were not quantitated. Comorbidity prevalence, including macrocephaly, is likely underestimated due to underdiagnosis.

In sum, >50% of patients with NF1 had no relative with NF1. CALMs and intertriginous freckling are by far the most common presenting cutaneous signs which would satisfy criteria for NF1 diagnosis. Despite high cutaneous burden, NF1 is a systemic disease requiring a multidisciplinary approach. Dermatologist awareness of NF1 extracutaneous manifestations is important for patient referral to appropriate specialties and for counseling tailored to their unique needs.

## Conflicts of interest

Dr Lipner has served as a consultant for Ortho-Dermatologics, Eli Lilly, BelleTorus Corporation, and Moberg Pharmaceuticals. Authors Curtis, De Barros, Albucker, and Qiu have no conflicts of interest to declare.
